# Changes in COVID-19 vaccine hesitancy at different times among residents in Guangzhou, China

**DOI:** 10.3389/fpubh.2023.1164475

**Published:** 2023-06-13

**Authors:** Lu Chen, Zhi Li, Xiaoxiao Lu, Yibin Deng, Katie Lu, Tiegang Li, Ling Lu, Zhiwei Wang, Jiachun Lu

**Affiliations:** ^1^The State Key Lab of Respiratory Diseases, Institute of Public Health, Guangzhou Medical University, Guangzhou, China; ^2^Department of English and American Studies, Faculty of Languages and Literatures, Ludwig Maximilian University (LMU), Munich, Germany; ^3^Centre for Medical Laboratory Science, The Affiliated Hospital of Youjiang Medical University for Nationalities, Baise, Guangxi, China; ^4^Department of Disease Prevention and Control, Guangzhou Municipal Health Commission, Guangzhou, China; ^5^Department of Hotline Management, Guangzhou Center for Disease Control and Prevention, Guangzhou, China

**Keywords:** COVID-19 vaccine, vaccine hesitancy, influencing factors, vaccination rate, immunization

## Abstract

**Background:**

Vaccination as a fundamental pillar of promoting public health and interest is critical to limiting the COVID-19 pandemic. However, many citizens are still hesitant about this epidemic prevention measure. This article aimed to understand the COVID-19 vaccination and hesitancy rates among Guangzhou residents at different points in time as well as to explore the relevant factors that cause vaccination hesitancy.

**Methods:**

We conducted a total of nine cross-sectional surveys by enrolling 12,977 questionnaires among Guangzhou residents through the online survey software called “WenJuanXing” between April 2021 and December 2022, and residents made their choices by judging their willingness to vaccinate. These surveys collected data on the participants' sociodemographic characteristics, vaccination status, vaccine hesitancy, and factors influencing this hesitancy. The Chi-squared test was used for univariate analysis and the multivariate logistic regression model was used to further adjust the influence of the confounding factors to evaluate the main factors affecting the hesitancy of the COVID-19 vaccine at different periods.

**Results:**

Over the course of 2021–2022, a total of 12,977 residents in the study area were surveyed. The vaccine hesitancy rates fluctuated over time. From April to June 2021, the vaccine hesitancy rate decreased from 30% to 9.1% and then increased to 13.7% in November. However, from April to December 2022, the hesitancy rate continued to rise from 13.4% to 30.4%. Vaccination rates, the epidemic waves of COVID-19, and changes in policies may all be possible factors that contributed to these fluctuations in vaccine hesitancy rates. We found statistically significant correlations between factors, such as residence, education, and occupation, and vaccine hesitancy at certain points of time. The results of the surveys in April and June 2021 showed that rural residents showed higher vaccine hesitancy rate than urban residents. Their lower education level was associated with higher vaccine hesitancy. Workers and farmers are more likely to have vaccine hesitancy than people with other occupations. The univariate analysis showed that people with underlying medical conditions and lower perceived health status were more likely to experience vaccine hesitation. Logistic regression analysis revealed that the health status of individuals is the most important factor leading to vaccine hesitancy, and residents' underestimation of domestic risks and overconfidence in personal protection measures were also contributing factors. At different stages, vaccine hesitancy among residents was related to vaccine side effects, safety and efficacy, convenience fluctuation, and various factors.

**Conclusion:**

In the present study, we found that vaccine hesitancy did not display a consistent downward trend but it fluctuated over time. Higher education, residing in urban areas, lower perceived disease risk, and concerns about the vaccine's safety and side effects were risk factors for vaccine hesitancy. Implementing appropriate interventions and educational programs tailored to address these risk factors may prove to be effective in enhancing public confidence on vaccination.

## Introduction

Severe acute respiratory syndrome coronavirus-2 (SARS-CoV-2), which caused the COVID-19 pandemic in December 2019, has caused great harm to the physical and mental health of people as well as incurred financial losses around the world (1). In addition to preventive measures (such as social distancing, washing hands, and wearing face-masks) and treatment, vaccination has been proven to be an effective tool for managing public health as essential to curbing the spread of SARS-CoV-2/COVID-19 (2). Research has shown that (3), for a COVID-19 vaccine, assuming that the prescribed vaccine has the highest possible efficacy, the vaccination rate of the general public must be above 70% to achieve herd immunity (4). To achieve herd immunity, a sufficient proportion of the population must be vaccinated to prevent the spread of diseases among the population. Therefore, the willingness of the public to vaccinate is decisive in achieving herd immunity (5).

On 30 December 2020, the State Food and Drug Administration of China approved the conditional launch of Sinopharm's COVID-19 inactivated vaccine (6). However, with the launch of the COVID-19 vaccine, various negative reports concerning the vaccine have also been propagated on the internet, making people hesitant about the vaccine and even refusing to be vaccinated (7). According to the survey, public trust in vaccination has declined globally, for both COVID-19 and other vaccines, in general, leading to an increase in vaccine hesitancy (8, 9). Vaccine hesitancy was listed by the WHO in 2019 as one of the top ten threats to global health (10). There are many reasons for vaccine hesitancy, including a lack of knowledge about the importance and necessity of vaccines, doubts about vaccine safety and efficacy, mistrust of health providers and vaccine strategists, availability of vaccination, geographic location, and concerns for costs (11).

Vaccination rates may vary by time, region, and specific brand of vaccine; however, vaccine hesitancy will lead to a decline in overall vaccination rate inevitably, making it impossible to achieve or maintain herd immunity to protect those with vaccine contraindications or individuals who failed to develop an immune response (12). Declining vaccination rates increase the risk of vaccine-preventable disease outbreaks (13). An online survey of people over the age of 18 in France found that 26% of respondents would refuse a vaccine even if it were available (14). In surveys conducted in the UK and Canada, 9 and 14%, respectively, of respondents refused to be vaccinated against COVID-19 (15, 16). According to a national cross-sectional survey report by Peking University, 67.1% of participants in China are willing to receive the COVID-19 vaccine, while 9.0% of the participants declined vaccination altogether and 35.5% of participants reported vaccine hesitancy (5). Even with guaranteed vaccine availability, it is still challenging to convince a sufficient number of individuals to receive the vaccine due to people's hesitation about vaccines (17). A recent survey conducted among healthcare professionals and students identified fear of unforeseen future effects as the main reason for their hesitation to vaccinate (18). Additional studies exploring the acceptability of the anti-SARS-CoV-2 vaccine co-administered with the influenza vaccine found that healthcare workers were more motivated than other groups to receive the vaccine, and the anti-SARS-CoV-2 vaccine was more acceptable when administered in combination with the influenza vaccine (19).

Although the Chinese COVID-19 vaccination rate has exceeded 80%, the mutation of the virus remains a concern as studies have shown that people can still contract SARS-CoV-2 even after completing the complete vaccination process. Therefore, investigating vaccine hesitancy and its influencing factors is crucial to prevent another outbreak of the disease and ensure high vaccination rates for booster doses (4). Tang et al. (20) explored the protective effect of the vaccine against COVID-19 pneumonia caused by virus mutation in Henan Province. The results demonstrated that the protective effect of the vaccine began to decline 6 months after the initial vaccination, but the protective effect was restored through homologous vaccination upon vaccine readministration (20). Evidence shows that, while COVID-19 vaccines are less effective in preventing the spread of the disease, vaccines are effective in preventing symptomatic SARS-CoV-2 infection from developing into severe stages (21–23). From 2021 to 2022, we investigated changes in COVID-19 vaccine hesitancy and vaccination status among 12,977 residents in Guangzhou across different periods and analyzed the relevant factors leading to vaccine hesitancy. This main aims of the study are to provide guidelines for the government to develop targeted vaccination programs, reduce the rate of vaccine hesitation, and increase the acceptance and enthusiasm for vaccination among the population.

## Materials and methods

### Study design

We conducted a population-based cross-sectional online survey between April 2021 to December 2022 aimed at assessing the COVID-19 vaccine hesitancy and its influencing factors among Guangzhou residents. Nine rounds of surveys were conducted in this study and distributed to all residents living in Guangzhou. The questionnaire includes three aspects: demographic characteristics, COVID-19 vaccination status and hesitancy, and influencing factors. This study has a non-duplicate cross-sectional survey that randomly sampled mobile phone numbers registered in Guangzhou. The staff of the Guangzhou Center for Disease Control and Prevention sent questionnaires to residents' mobile phones through WeChat, SMS, and other forms from the 1st to the 15th of each month during the survey period. The participants were required to complete the survey questionnaires through mobile phones. For those residents who have already filled out questionnaires, they were be given questionnaires in the subsequent survey. Data were collected on Wen Juan Xing (like Amazon Mechanical Turk, Qualtrics, SurveyMonkey, or CloudResearch), which provides the function of designing questionnaires and surveys online.

### Calculation of sample size

According to the formula for calculating the sample size of enumerating data from the current situation survey in epidemiological studies,


$$\begin{array}{l} \text{N }=\text{ p}\times \text{q}/\text{S}{\text{p}}^{2}, \end{array}$$

where *N* represents the sample size required for the investigation, *p* is the expected positive rate or prevalence rate of the investigation, q = 1-p, S_p_ = d/ Z_α_, *d* is the allowable error, *z* is the boundary value of the standard normal distribution, and *Z*_α_ is the significance test statistic. For instance, when α = 0.05, Z_α_ = 1.96, and d = 0.1p is generally used, the formula for calculating the sample size can be rewritten as:


$$\begin{array}{l} \text{N }=\;400\times \text{q}/\text{p} \end{array}$$

Based on the available data, the ratio of the herd immunity level (pc) required to prevent transmission for a vaccine with 100% efficacy and lifelong protection is (1-1/R_0_) in the population, where R_0_ represents the basic reproductive number (24). Given that most countries had pre-lockdown R_0_ values between 2.5 and 3.5, the required herd immunity would be approximately 60–72% (25). Since the vaccine is unlikely to be 100% effective, much higher vaccination rates are needed to ensure herd immunity. Therefore, a minimum vaccination rate of above 60% is required, and this value is further impacted by vaccine efficacy. Therefore, *p* = 0.5 is substituted into the formula, and this yields a sample size of 400. However, in consideration of the large number of urban residents in the survey, their strong health awareness, and their high willingness to vaccinate, the sample size was doubled. In addition, the sample size of each sample should not be <800 people.

### Data collection

The nine questionnaires were completed by different people in a time series. We adopted the method of simple random sampling without replacement (i.e., each respondent had only one opportunity to participate) and randomly distributed the questionnaires to the mobile phone numbers registered by Guangzhou residents. Data were collected during the months of April to June and November in 2021 as well as April to June and November to December in 2022. We collected demographic data, including age, gender, place of residence, education, monthly income, and occupation, as well as information relating to vaccination status and vaccine hesitancy. We also investigated factors that contribute to vaccine hesitancy.

### Vaccine hesitancy

A report from the Strategic Advisory Group of Experts (SAGE) on immunization of the WHO defines vaccine hesitancy as “delay in acceptance or refusal of vaccination despite availability of vaccinations services. Vaccine hesitancy is complex and context specific, varying across time, place, and vaccines. It is influenced by factors such as complacency, convenience, and confidence (26).” Vaccine hesitancy is a continuum between complete acceptance and full rejection. This study divided vaccine hesitancy into seven categories: fully accepted, accepted but unsure, partially accepted, delayed vaccination, partially rejected, rejected but unsure, and completely rejected. Individuals who chose the two categories, fully accepted and accepted but unsure, were considered to have no vaccine hesitancy. Those who selected the other five were classified as having vaccine hesitancy. This classification provides a framework for understanding the various degrees of vaccine hesitancy that individuals may exhibit.

### Vaccine hesitancy rate

The vaccine hesitancy rate refers to the proportion of the total number of people who participated in the survey and were classified as having vaccine hesitancy toward COVID-19 vaccine during the survey period.

### Vaccination rate

The survey on vaccination rate examines both the vaccination status and the actual vaccination status of the survey participants. The vaccination rates are calculated based on the number of individuals who have received at least one dose of vaccine and those who have completed the full three-dose course (considered as fully vaccinated). The vaccination rate is the percentage of people who have been vaccinated out of the total number of people surveyed each month (the samples drawn each month are different). The actual cumulative vaccination rate of the population refers to the proportion of the cumulative number of vaccinated people during a particular month, relative to the permanent resident population of Guangzhou.

### Determinants of vaccine hesitancy

There are many potential reasons as to why the residents are hesitant to receive vaccination against COVID-19, including underlying diseases (chronic diseases such as hypertension and diabetes, and COPD), self-assessed health scores, unpleasant experiences with vaccination, consultation with professionals about COVID-19 vaccination, and less knowledge of vaccines (including whether the mutant strain affects the effectiveness of the vaccine in preventing disease and whether vaccination reduces the likelihood of developing severe stage in the future).

### Knowledge and beliefs about vaccination

To investigate residents' knowledge and beliefs about vaccination and the influencing factors of vaccine hesitancy, we designed 18 questions from three aspects, namely, self-confidence, complacency, and convenience. Self-confidence includes trust in the efficacy and safety of vaccines, reliability and competence of health services and health professionals, and motivations of vaccine decision-makers. Complacency is mainly due to the lack of sufficient understanding of the necessity and importance of vaccines. Convenience mainly includes vaccine availability, geographic location, and willingness to pay. Each item is scored on a scale of “1 = strongly disagree” to “5 = strongly agree” with reference to the Likert scale. The higher the score, the greater the impact this factor has on residents' hesitation in receiving the COVID-19 vaccine. We tested the reliability and validity of the scale, respectively, and found that the Cronbach's α values in April 2021, May 2021, and June 2021 were 0.667, 0.736, and 0.876. Overall results with values >0.6 were accepted.

### Statistical analysis

Descriptive analyses of demographic characteristics were performed. Categorical variables are expressed as the number of cases and percentages (%) in a different group. The survey participants were divided into four categories according to their age, namely, <25, 25–34, 35–44, and >45 years. The subjects were divided into the vaccine-hesitant group and the vaccine non-hesitant group according to the above definition of vaccine hesitancy. The vaccine hesitancy rate for each month was calculated individually. Univariate analysis was performed on the data from April to June 2021 using the chi-squared test. Internal consistency of the scale scores was evaluated with Cronbach's α. The vaccine-hesitant individuals were regarded as the case group and the vaccine non-hesitant individuals as the control group, and the odds ratio (OR) and 95% confidence interval (CI) were calculated by the binary logistic regression model. A two-sided test was used for statistical analysis in this study, and the test level was α = 0.05. Taking “1 = strongly disagree” as a reference, for each increment of 1, the risk of vaccine hesitancy due to this factor increases OR times.

## Results

A total of 15,000 questionnaires were distributed during the course of this survey, of which 13,521 of them were completed. We performed quality control on the questionnaires, excluding questionnaires with missing information and questionnaire completion time of <60 s. Finally, 12,977 valid questionnaires were collected, and the response rate was 95.9%.

### Demographic characteristics

Table 1 describes in detail the date of the survey, the number of people in each round of surveys, and the distribution of respondents' age, gender, region, education level, monthly income, and occupation. The population participating in this survey predominantly consists of individuals aged between 20 and 50 years. There are more urban residents than rural residents. In addition, most participants have attained an undergraduate degree or higher.

**Table 1:** Demographic characteristics.

**Characteristics**	**April 2021**	**May 2021**	**June 2021**	**November 2021**	**April 2022**	**May 2022**	**June 2022**	**November 2022**	**December 2022**
Total	1,000	1,023	1,101	1,577	1,945	1,162	1,350	1,620	2199
**Age (years)**							
<20	20 (2.0%)	22 (2.2%)	18 (1.6%)	64 (4.1%)	45 (2.3%)	30 (2.6%)	16 (1.2%)	18 (1.1%)	97 (4.4%)
20–35	669 (66.9%)	582 (56.9%)	613 (55.7%)	769 (48.8%)	994 (51.1%)	633 (54.5%)	741 (54.9%)	937 (57.8%)	1115 (50.7%)
35–50	251 (25.1%)	331 (32.4%)	351 (31.9%)	530 (33.6%)	744 (38.3%)	375 (32.3%)	503 (37.3%)	576 (35.6%)	755 (34.3%)
>50	60 (6.0%)	88 (8.6%)	119 (10.8%)	214 (13.6%)	162 (8.3%)	124 (10.7%)	90 (6.7%)	89 (5.5%)	232 (10.6%)
**Gender**							
Men	441 (44.1%)	433 (42.3%)	472 (42.9%)	801 (50.8%)	1055 (54.2%)	682 (58.7%)	599 (44.4%)	519 (32.0%)	1249 (56.8%)
Women	559 (55.9%)	590 (57.7%)	629 (57.1%)	776 (49.2%)	890 (45.8%)	480 (41.3%)	751 (55.6%)	1337 (82.5%)	950 (43.3%)
**Residence**							
Rural	200 (20.0%)	160 (15.6%)	200 (18.2%)	285 (18.1%)	360 (18.5%)	182 (15.7%)	196 (14.5%)	283 (17.5%)	448 (20.4%)
Urban	800 (80.0%)	863 (84.4%)	901 (81.8%)	1292 (81.9%)	1585 (81.5%)	980 (84.3%)	1154 (85.5%)	1337 (82.5%)	1751 (79.6%)
**Education**							
Elementary school and below	23 (2.3%)	14 (1.4%)	14 (1.3%)	11 (0.7%)	19 (1.0%)	2 (0.2%)	7 (0.5%)	5 (0.3%)	16 (0.7%)
Junior high school and high school	213 (21.3%)	257 (25.1%)	197 (19.9%)	361 (22.9%)	366 (18.8%)	153 (13.2%)	173 (12.8%)	186 (11.4%)	488 (22.2%)
Undergraduate and above	764 (76.4%)	752 (73.5%)	890 (80.8%)	1205 (76.4%)	1560 (80.2%)	1007 (86.7%)	1170 (86.7%)	1429 (88.2%)	1695 (77.1%)
**Monthly income**							
<5,000	510 (51.0%)	257 (25.1%)	297 (27.0%)	556 (35.3%)	543 (27.9%)	254 (21.9%)	288 (21.3%)	401 (24.8%)	662 (30.1%)
5,000~10,000	307 (30.7%)	553 (54.1%)	457 (41.5%)	589 (37.3%)	779 (40.1%)	407 (35.0%)	511 (37.9%)	638 (39.4%)	855 (38.9%)
>10,000	183 (18.3%)	213 (20.8%)	347 (31.5%)	432 (27.4%)	623 (32.0%)	501 (43.1%)	551 (40.8%)	581 (35.8%)	682 (31.0%)
**Profession**							
Healthcare workers	54 (5.4%)	47 (4.6%)	30 (2.7%)	77 (4.9%)	46 (2.4%)	44 (3.8%)	64 (4.7%)	96 (5.9%)	62 (2.8%)
Administrators, staff, cultural educators,	298 (29.8%)	473 (46.2%)	671 (60.9%)	811 (51.4%)	1044 (53.7%)	716 (61.6%)	795 (58.9%)	993 (61.4%)	1154 (52.5%)
Businesspersons, and service workers	246 (24.6%)	140 (13.7%)	47 (4.3%)	94 (6.0%)	137 (7.0%)	47 (4.0%)	94 (7.0%)	61 (3.8%)	195 (8.9%)
Workers and farmers	34 (3.4%)	83 (8.1%)	53 (4.8%)	96 (6.1%)	138 (7.1%)	48 (4.1%)	43 (3.2%)	50 (3.1%)	181 (8.2%)
Others^*^	368 (36.8%)	280 (27.4%)	300 (27.2%)	499 (31.6%)	580 (29.8%)	307 (26.4%)	354 (26.2%)	420 (25.9%)	607 (27.6%)

^*^students, housewives, unemployed individuals, retired workers, and others.

### Vaccine hesitancy rate

As shown in Table 2, between April 2021 and June 2021, the vaccine hesitancy rate showed a significant downward trend, i.e., from 30% to the lowest value of 9.1%. However, in the November 2021 survey, we found a small increase in the vaccine hesitancy rate (13.7%). The survey showed that there has been a continuous upward trend in the vaccine hesitancy rates (five consecutive surveys from April 2022 to December 2022), which peaked to 30.4% in December 2022.

**Table 2:** Vaccine hesitancy rate.

**Date**	**Total**	**Number of people who are vaccine hesitant**	**Vaccine hesitancy~rate (95%CI)**
April 2021	1,000	300	30.0% (27.2~32.8)
May 2021	1,023	139	13.6% (11.5~15.7)
June 2021	1,101	100	9.1% (7.4%~10.8)
November 2021	1,577	216	13.7% (12.0~15.4)
April 2022	1,945	261	13.4% (11.9~14.9)
May 2022	1,162	229	19.7% (17.4~22.0)
June 2022	1,350	310	23.0% (20.6~25.2)
November 2022	1,620	384	23.7% (21.6~25.8)
December 2022	21,99	669	30.4% (28.5~32.3)

### Factors influencing vaccine hesitancy

#### COVID-19 vaccination rate

**Figure 1:**
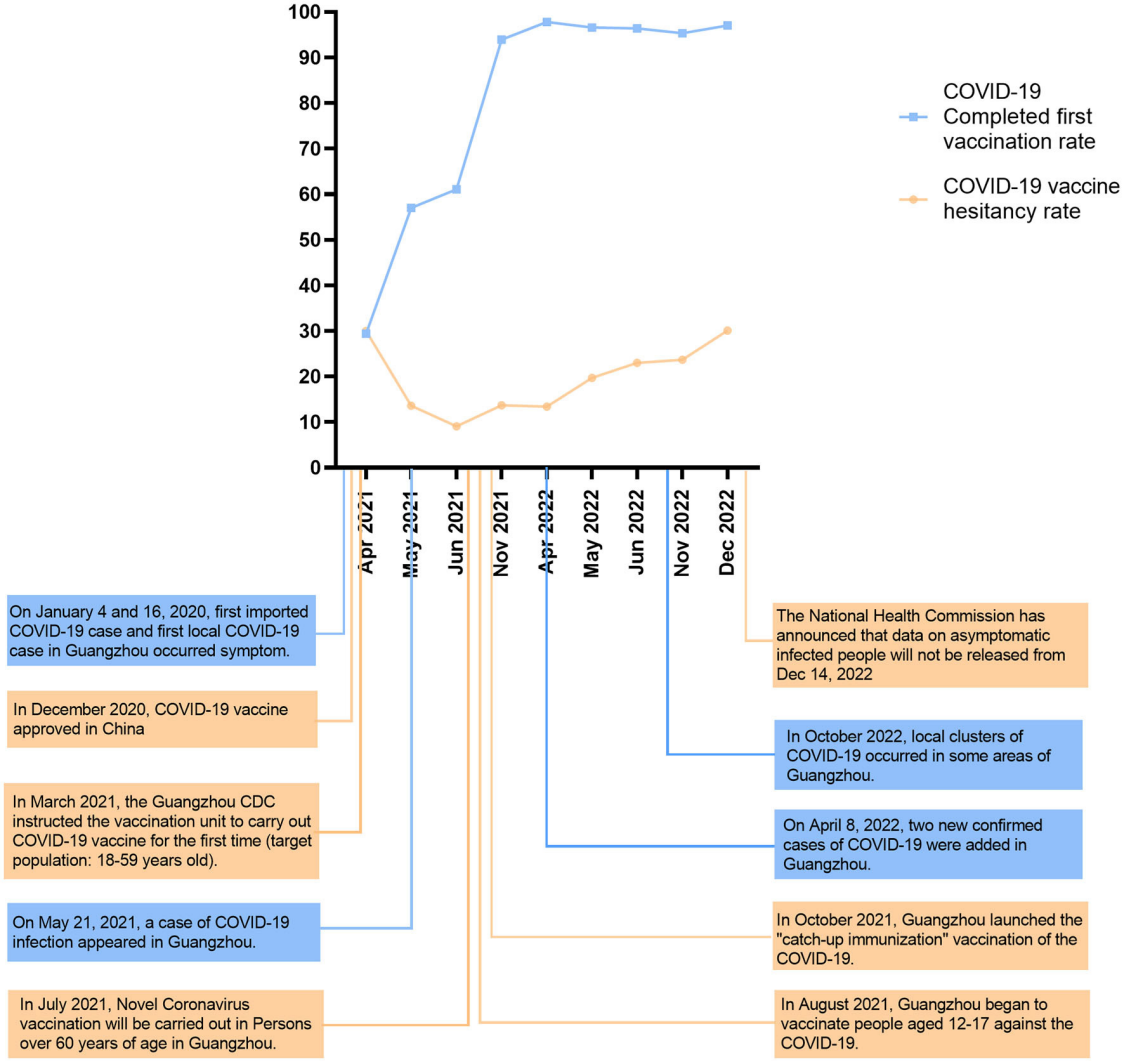
COVID-19 vaccination rate and hesitation rate among Guangzhou residents at different stages. The vaccination policy issued by Guangzhou city and the fluctuation of the epidemic situation.

Since March 2021, when the Guangzhou Center for Disease Control and Prevention first announced its COVID-19 vaccination program, the vaccination rate in Guangzhou has shown an obvious upward trend. The survey shows a sharp increase in the number of people vaccinated against COVID-19 from April to June 2021, according to Guangzhou residents' vaccination data provided by the Guangzhou Municipal Health Commission. The data, including the vaccination rate for at least one dose and the full course of vaccination, reveal that the vaccination rate has reached more than 90% in November 2021 (Table 3). It is consistent with the results of our investigation, indicating that this study is representative. As shown in Figure 1, the chi-squared test revealed that the *P*-values of the survey results at each stage were all <0.001, indicating that there was a statistically significant difference between the two groups in vaccination.

**Table 3:** COVID-19 vaccination rate in Guangzhou.

**Date**	**Vaccination rate in subjects**		**Actual cumulative vaccination rate in the population**	
	**Completed first vaccination**	**Completed full vaccination**	**Completed first vaccination**	**Completed full vaccination**
April 2021	29.4%	—	20.2%	—
May 2021	57.1%	—	51.0%	—
June 2021	61.1%	—	59.6%	—
November 2021	93.9%	11.6%	90.9%	11.2%
April 2022	97.8%	83.9%	94.6%	77.3%
May 2022	96.6%	78.7%	95.0%	81.5%
June 2022	96.4%	80.7%	95.3%	81.9%
November 2022	95.3%	78.6%	96.4%	84.0%
December 2022	97.0%	84.6%	96.5%	84.3%

#### Epidemic wave of COVID-19 in Guangzhou

**Figure 2:**
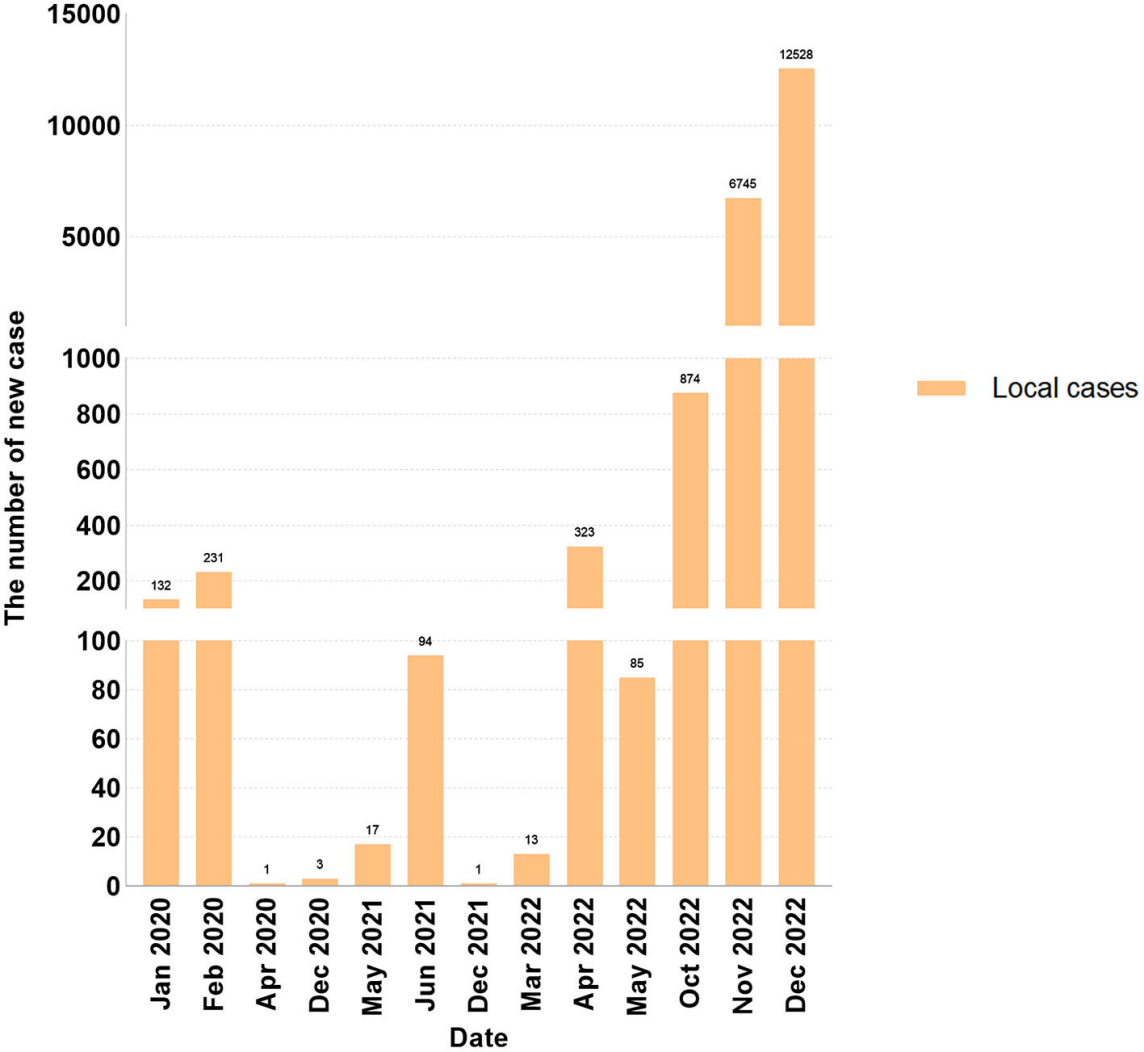
The new local COVID-19 cases report in Guangzhou form January 2021 to June 2022 (as the national health ccommission announced that data will not be released from December 14, 2022, the data was collected until December 21, 2022).

Since the emergence of COVID-19, Guangzhou has experienced a total of five local waves of the epidemic. The first wave occurred from January to April 2020, followed by the second wave from May to June 2021, during when the *Delta* virus variant was prevalent. The third wave was from March to May 2022, when the virus variant was *Omicron*. The fifth wave emerged from October to December 2022 (Figure 2). The correlation coefficients between the vaccine hesitancy rate and the number of cases during the second, third, and fourth waves were r_1_ = -0.783, r_2_ = 0.996, and r_3_ = 1.000.

#### Sociodemographic factors

In the April 2021 survey, individuals living in urban areas (31.6%) were more likely to develop vaccine hesitancy than those living in rural areas (23.6%). However, in the subsequent May and June surveys, residence could no longer be considered a statistically significant factor associated with vaccine hesitancy. In both the April and June 2021 surveys, people with primary school education and below showed higher rates of hesitation. and the results were statistically significant. In the May 2021 survey, healthcare workers exhibited no vaccine hesitancy, while workers and farmers had a higher likelihood of vaccine hesitancy than individuals did in other occupations. The results of the May–June 2021 surveys revealed that there are more negative attitudes toward vaccination among those with underlying medical conditions. After examining the self-assessed health scores, three different outcomes emerged. The results of the April 2021 survey showed that residents with moderate self-assessed health were more likely to be hesitant toward vaccinations. As time went on, however, hesitation increased significantly among residents who self-assessed their health as poor in June, following adverse side effects and various negative reports concerning the vaccine. The June study also found that people with unpleasant past vaccination experiences were more likely to be hesitant to get vaccinated again, and, as evidenced by the April study, those who did not believe that the COVID-19 vaccine would reduce COVID-19 symptoms were more likely to be hesitant to vaccinate (Table 4).

**Table 4:** Determinants of vaccine hesitancy.

	**April 2021**		**May 2021**		**June 2021**	
	**Vaccine hesitancy rate(95%CI)**	***P*** **-value**	**Vaccine hesitancy rate(95%CI)**	***P*** **-value**	**Vaccine hesitancy rate(95%CI)**	***P*** **-value**
**Age (years)**		0.995		0.234		0.616
<20	30.0% (9.9~50.1)		27.3% (8.7~45.9)		16.7% (-0.5~33.9)	
20~	29.7% (26.3~33.2)		13.7% (10.9~16.5)		8.8% (6.6~11.1)	
35~	30.7% (25.0~36.4)		12.1% (8.6~15.6)		9.7% (6.6~12.8)	
50~	30.0% (18.4~41.6)		14.8% (7.4~22.2)		7.6% (2.8~12.3)	
**Gender**		0.922		0.281		0.060
Men	30.2% (25.9~34.4)		12.2% (9.2~15.3)		7.2% (4.9~9.5)	
Women	29.9% (26.1~33.7)		14.6% (11.7~17.4)		10.5% (8.1~12.9)	
**Residence**		**0.028**		0.491		**0.556**
Rural	23.6% (17.7~29.5)		11.9% (6.9~16.9)		8.0% (4.2~11.8)	
Urban	31.6% (28.0~34.4)		13.9% (11.6~16.2)		9.3% (7.4~11.2)	
**Education**		**0.030**		0.325		**0.017**
≤Primary school	52.2% (31.8~72.6)		14.3% (-4.0~32.6)		28.6% (4.9~52.2)	
Junior high school and high school	32.9% (26.6~39.2)		16.3% (11.8~20.9)		11.2% (6.8~15.6)	
≥Undergraduate	28.5% (25.3~31.7)		12.6% (10.3~15.0)		8.3% (6.5~10.1)	
**Monthly income**		0.535		0.792		0.254
<5,000	28.4% (24.5~32.3)		14.8% (10.4~19.1)		10.8% (7.2~14.3)	
5,000~10,000	31.9% (26.7~37.1)		13.6% (10.2~15.8)		7.4% (5.0~9.8)	
>10,000	31.1% (24.4~37.9)		13.6% (9.0~18.2)		9.8% (6.7~12.9)	
**Profession**		0.203		**0.010**		0.637
Healthcare workers	20.4% (9.6~31.1)		0.0%		6.7% (9.6~31.1)	
Administrators, staff, cultural educators,	27.5% (22.4~32.6)		14.4% (11.2~17.5)		8.2% (22.4~32.6)	
Businesspersons, and service workers	30.5% (24.7~36.2)		10.7% (5.6~15.8)		12.8% (24.7~36.2)	
Workers and farmers	35.3% (19.2~51.4)		21.7% (12.8~30.6)		9.4% (19.2~51.4)	
Others^*^	32.6% (27.8~37.4)		13.6% (9.6~17.6)		10.7% (27.8~37.4)	
**Underlying diseases (chronic diseases such as hypertension and diabetes)**		0.175		**<0.001**		**0.001**
Yes	37.7% (25.5~49.9)		45.3% (33.1~57.5)		18.7% (10.7~26.7)	
No	29.5% (26.6~32.4)		11.5% (9.5~13.5)		8.2% (6.5~9.9)	
**Self-assessed health score**		**0.032**		**0.010**		**<0.001**
Good	29.5% (26.2~32.9)		12.7% (10.6~14.9)		7.8% (6.2~9.5)	
moderate	40.4% (31.0~49.8)		25.4% (15.2~35.5)		17.3% (9.9~24.8)	
Poor	25.8% (19.4~32.3)		0.0%		71.4% (38.0~104.9)	
**Have you had an unpleasant experience during the vaccination process?**		0.899		0.106		**0.001**
Yes	29.5% (21.4~37.6)		20.7% (10.3~31.1)		22.4% (10.8~34.1)	
No	30.1% (27.0~33.1)		13.2% (11.1~15.3)		8.5% (6.8~10.1)	
**Have you consulted a professional for the COVID-19 vaccine?**		0.177		0.160		0.995
Yes	27.1% (22.2~32.0)		15.7% (13.3~21.7)		9.1% (6.1~12.1)	
No	31.3% (27.9~34.8)		12.5% (10.0~15.0)		9.1% (7.0~11.1)	
**Do you think the emergence of mutant strains has an impact on vaccines?**		0.519		0.275		0.316
Yes	28.8% (24.1~33.4)		11.7% (8.8~14.6)		10.4% (7.8~13.0)	
No	30.7% (27.1~34.3)		15.4% (11.3~19.6)		8.0% (3.0~13.0)	
I don't know.	—		14.9% (10.6~19.2)		7.8% (5.3~10.2)	
**Do you think getting the COVID-19 vaccine can reduce the symptoms of COVID-19 in the future?**		**0.000**		0.319		**<0.001**
Yes	25.5% (22.3~28.7)		12.6% (10.1~15.0)		6.5% (4.8~8.2)	
No	45.7% (31.3~60.0)		15.7% (5.7~25.7)		39.5% (23.9~55.0)	
I don't know.	40.2% (34.1~46.4)		16.2% (11.6~20.8)		12.6% (8.6 ~16.7)	

^*^students, housewives, unemployed individuals, retired workers, and others.

#### Influencing factors of vaccine hesitancy on knowledge and belief

**Figure 3:**
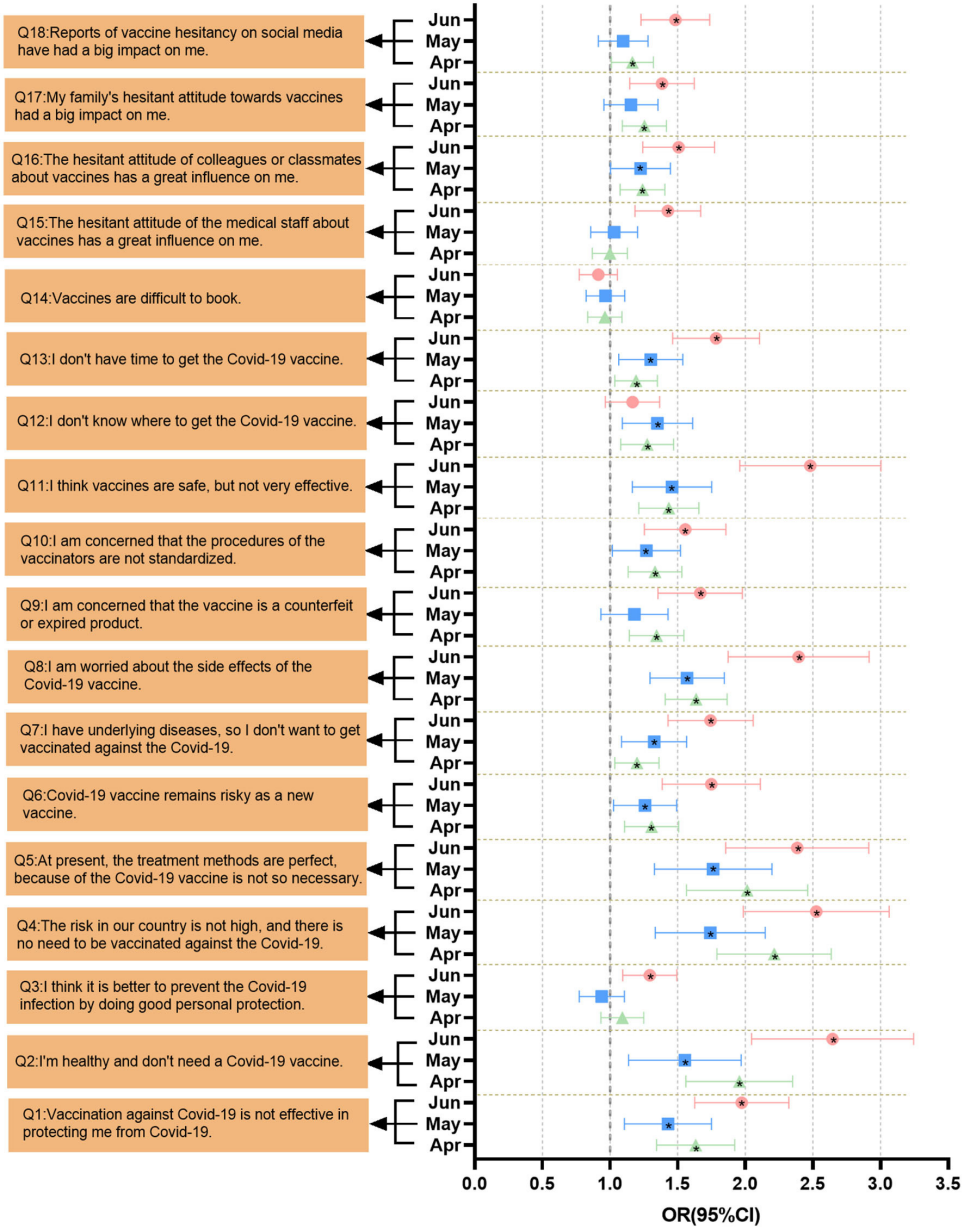
Multivariate logistic regression analysis of factors associated with COVID-19 vaccine hesitancy. The OR value and 95% CI were obtained after adjusting for gender and age. Graphs in black with “*” inidcate that the item is satistically significant.

Figure 3 shows the adjusted odds ratios and 95% confidence intervals for factors that contributed to vaccine hesitancy between April 2021 and June 2021. Among these 18 influencing factors, Q1–Q5 belong to complacency, Q6–Q11 and Q15–Q18 belong to confidence, and Q12–Q14 belong to convenience. The main influencing factors of vaccine hesitancy among individuals in April, May, and June 2021 are given as follows: a lower perceived risk of contracting COVID-19 in China (April: OR = 2.191, 95% CI = 1.809–2.653; May: OR = 1.703, 95% CI = 1.347–2.153; and June: OR = 2.441, 95% CI = 1.972–3.020), sufficiency with existing treatments (April: OR = 1.978, 95% CI = 1.583–2.470; May: OR = 1.722, 95% CI = 1.344–2.206; and June: OR = 2.280, 95% CI = 1.830–2.842), and disallowing themselves of the need for vaccination due to their current health status (April: OR = 1.925, 95% CI = 1.573–2.356; May: OR = 1.506, 95% CI = 1.152–1.969; and June: OR = 2.545, 95% CI = 2.029–3.192). However, all three items belong to the complacency factor. In addition to these three influencing factors, the second related factor leading to vaccine hesitancy is the fear of vaccine side effects (April: OR = 1.631, 95% CI = 1.417–1.876; May: OR = 1.560, 95% CI = 1.310–1.857; and June: OR = 2.327, 95% CI = 1.876–2.886). There was no statistically significant difference between April and June 2021 in terms of vaccine availability. The following factors all led to vaccine risk hesitation among individuals during the 3 months of the survey: vaccines cannot effectively prevent new coronavirus infection (April: OR = 1.608, 95% CI = 1.349–1.917; May: OR = 1.416, 95% CI = 1.131–1.772; and June: OR = 1.983, 95% CI = 1.664–2.363), b vaccines are risky (April: OR = 1.314, 95% CI = 1.129–1.529; May: OR = 1.250, 95% CI = 1.039–1.505; and June: OR = 1.718, 95% CI = 1.399–2.109), worries that vaccination personnel are not standardized (April: OR = 1.309, 95% CI = 1.128–1.519; May: OR = 1.265, 95% CI = 1.038–1.541; June: OR = 1.520, 95% CI = 1.255–1.841), the vaccine effectiveness is not high (April: OR = 1.425, 95% CI = 1.221–1.663; May: OR = 1.443, 95% CI = 1.180–1.764; and June: OR = 2.403, 95% CI = 1.952–2.958), lacked the time to vaccinate (April: OR = 1.181, 95% CI = 1.035–1.348; May: OR = 1.267, 95% CI = 1.059–1.516; and June: OR = 1.706, 95% CI = 1.431–2.034), and an hesitant attitude toward vaccine if colleagues or classmates were hesitant (April: OR = 1.230, 95% CI = 1.077–1.405; May: OR = 1.222, 95% CI = 1.021–1.464; and June: OR = 1.498, 95% CI = 1.257–1.785). Two factors were newly added in June 2021 as contributing factors to vaccine hesitancy, namely the belief that personal protection protects against COVID-19 (OR = 1.271, 95% CI = 1.091–1.481) and vaccine hesitancy among healthcare workers (OR = 1.418, 95% CI = 1.197–1.680).

## Discussion

This study highlights the importance of understanding the dynamic changes in residents' hesitancy since the COVID-19 vaccination program began in Guangzhou at the end of March 2021 and explored the main factors leading to vaccine hesitancy to propose targeted measures to increase residents' confidence and willingness to vaccinate and increase vaccination rates, particularly during times of increased risk or changes in vaccination policies. The attitudes and factors influencing vaccination hesitancy are not constant and may change over time and according to circumstances (27). For example, research in the United States showed that the hesitancy rates for a COVID-19 vaccine fell from 46.0% at the start of the survey to 35.2% 3 months after the survey (28). Conversely, in Hong Kong, the vaccination hesitancy rate during the two waves of the epidemic in February and August 2020 increased from 55.8 to 65.2% (29). Another long-term cross-sectional survey in Hong Kong reported that, along with the fluctuation of the coronavirus epidemic, people's hesitation concerning vaccination also showed fluctuation at different stages (30). Similar to the fluctuation in the previous study, the vaccine hesitancy rate among Guangzhou residents dropped from 30% to 9.1% between April and June 2021. However, in the November 2021 survey, we found a slight increase in vaccine hesitancy (13.7%) rather than a sustained decline. The Guangzhou Center for Disease Control and Prevention issued notices on vaccination for residents aged 12–17 years and over 60 years old from June to November 2021. Considering that children and the older adult are more likely to have side effects (such as dizziness, fatigue, etc.) owing to their poor physical fitness (31), this may have contributed to the increase in vaccine hesitancy in November. Since 8 April 2022, Guangzhou has ushered in a new wave of SARS-CoV-2 infections, which primarily infected vaccinated individuals. This may have caused vaccine hesitancy to increase from 13.4% to 23.0% between April and June 2022. Later, the fourth wave of the epidemic in Guangzhou in October 2022, and the alterations in various epidemic prevention and control policies may have caused residents to be lax in adhering to epidemic prevention and control policies and may have further affected vaccine hesitation. According to the survey, the vaccine hesitancy rate of Guangzhou residents reached 30.4% in December 2022, which was higher than the rate recorded at the beginning of the survey.

The residents' knowledge of vaccines was insufficient, such as failing to acknowledge that the COVID-19 vaccine can effectively mitigate the severity and mortality of COVID-19 (32) or having misconceptions such as believing that vaccination can completely prevent the infection of the disease. In light of this, we should actively educate the public, popularize vaccine-related knowledge, and disseminate correct vaccination information. Our research also found that unpleasant vaccination experiences and distrust of healthcare personnel (who administers vaccines) were also the key factors leading to hesitancy. Therefore, during the vaccination process, we should conduct uniform and standardized training for staff to alleviate residents' doubts concerning the professionalism of vaccination of those administering vaccines to reduce the public's resistance (5).

Since the organization and implementation of the booster vaccination program in Guangzhou in October 2022, more than 80% of the residents completed the full course of vaccination in November of the same year, but the situation of vaccine hesitancy continues to persist. The extent of vaccination hesitation cannot be fully reflected by the vaccination rate (33). Moreover, we found that a small percentage of residents who had received the first dose of vaccination had not completed the full course of vaccination. Consequently, addressing and overcoming residents' hesitancy remains an inevitable and ongoing challenge.

Our survey coincides with the timing of the second, third, and fourth waves of the COVID-19 epidemic in Guangzhou. The obtained correlation coefficient obtained from our analysis of the survey from April to June 2021 showed that the vaccine hesitancy rate decreases with the emergence of the epidemic (r <0). However, the surveys taken in April–June 2022 and November–December 2022 showed that the correlation coefficient was positive (r>0), indicating that the epidemic has led to an increase in the rate of vaccine hesitancy. In May 2021, Guangzhou City launched its vaccination campaign for the first time, and with the assistance and extensive publicity effort from the state and government, residents began to vaccinate against COVID-19. The emergence of new COVID-19 cases that month may have sparked panic about the disease and increased the demand for vaccinations among residents, thereby reducing the vaccine hesitancy rates. In the third and fourth waves of the epidemic, even though most people have been fully vaccinated, some of them were still infected. This led to questions among some people who do not understand the characteristics of vaccine-preventable diseases to question the effectiveness of the vaccine, thereby increasing the vaccination hesitancy rate (11).

Residence and education are important factors affecting vaccination. Consistent with the findings of Israel and the UK (3, 34), people living in urban areas tend to have a higher hesitation rate, which may be related to factors such as population density, income, etc. Compared to rural areas, urban areas have better medical resources and protective measures in place. Studies have shown that people in urban areas are more susceptible to negative information about vaccination and thus show distrust of WHO, while people in rural areas tend to be more receptive to government's calls to action and arrangements (35). Attitudes toward vaccine hesitancy vary across occupations, which may be due to differing levels of medical knowledge and their varying needs for preventive measures (36). Medical workers, for instance, will have a more accurate understanding of vaccine knowledge, and the thus demand for vaccines will be higher (36). In contrast, farmers or workers may be more likely to refuse vaccination due to lower health literacy or perceived low risk of contracting a disease (37). Furthermore, chronic diseases were also associated with vaccination hesitancy, as evidenced by a cross-sectional survey conducted in Hong Kong (29). A survey of COVID-19 vaccine hesitancy among chronically ill children aged 5–11 years in Italy showed that 26.3% of chronically ill patients were highly hesitant. The low perceived risk of children being infected with SARS-CoV-2 coupled with the fact that the children had not yet been vaccinated further exacerbated the degree of vaccine hesitancy (38). However, one particular finding of our study was that people with moderate self-rated health scores exhibited higher rates of vaccination hesitancy. This suggests that refusing vaccination because of physical health is also a major factor leading to hesitation.

Since the outbreak of the COVID-19 epidemic in late 2020, China has implemented a series of stringent measures and public health interventions to control the spread of COVID-19 (39, 40), which has also caused some members of the public to underestimate the risk of the disease (5). Our study found that the main reasons for refusing vaccination include: confidence in one's own robust immune system, confidence in the treatment of the disease, and the belief that existing preventive measures are sufficient to prevent COVID-19 without vaccination. Numerous studies have demonstrated that the perceived risk of contracting the disease also affects the public's vaccination intentions (41–44). We must increase public awareness of the new coronavirus and underscore the importance and necessity of vaccination in order to reduce vaccine hesitation rates and maximize the number of people vaccinated.

Consistent with findings from other countries (29, 45, 46), concerns about the safety and side effects of vaccination are also one of the main reasons for vaccine hesitancy. COVID-19 is an emerging infectious disease, and uncertainty about a new vaccine may further heighten public concerns about vaccination (47). A cross-sectional study of people in Italy who had completed initial vaccination showed that those who reported reluctance and uncertainty about getting the booster shot were mainly concerned about vaccine safety (48). To enhance the public's confidence in vaccination, national or government authorities should regularly monitor and disclose scientific information concerning the safety of vaccines and conduct timely health education and communication to alleviate the public's concerns about vaccine safety and side effects (33). Social media dissemination of information about vaccine safety is as important as that of vaccine efficacy (49). Research by Betsch et al. (50) showed that disproportionally widespread negative coverage of vaccines on the Internet may increase people's distrust of vaccines. Therefore, healthcare authorities should strengthen the control over the dissemination of vaccine-related information on social media platforms and prevent the dissemination of information that denigrates or exaggerates the safety and effectiveness of vaccines (51). Social media can also play a positive role as timely dissemination of accurate information about the COVID-19 vaccines and effective control of fake news related to COVID-19 vaccines can eliminate public hesitation about vaccination and help increase confidence in vaccination (52, 53). Vaccines are more likely to be accepted if the information about vaccines is endorsed by medical professionals, as well as their family, friends, and colleagues (54). Other studies have also demonstrated that medical practitioners' advice on vaccination is more likely to be adopted by the public (55). Therefore, medical workers should establish a good relationship of trust with the population, thereby alleviating people's concerns and addressing vaccine hesitancy (56). Previous studies have shown that organizing health education among professionals on immunization strategies can have a positive effect on disease prevention and is essential for good adherence to vaccination (57). In response to challenges in vaccine access and distribution, such as not having time to get vaccinated and not knowing where to receive vaccination, China has taken various corresponding measures, such as mobile vaccination vehicles and setting up mobile vaccination locations in various communities. These measures have brought great convenience to residents and promoted the vaccination campaign.

The limitation of this study is that it is a cross-sectional study, which makes it difficult to infer the influence of an individual or a set of factors on vaccine-hesitant behavior. However, this study adopted a random sampling method for different populations to guarantee the representativeness of the sample. Although the study only conducted three surveys on knowledge and belief, the results were largely consistent. Self-report questionnaires are susceptive to subjective interpretation and bias, which may affect the reliability and validity of the survey results. Therefore, we still need more research studies to confirm our findings. The study cannot provide conclusive evidence of a causal relationship between changes in background factors (including changes in news coverage of COVID-19 vaccines and policies) and changes in public risk perception-related vaccine hesitancy. Therefore, the inferences drawn from this study must be considered tentative.

## Conclusion

In this study, we found that vaccine hesitancy did not exhibit a steady decline over time but it rather fluctuated. Risk factors for vaccine hesitancy included higher education, urban residency, a lower perceived risk of contracting the disease, and concerns about the vaccine's safety and side effects. Appropriate interventions and education initiatives would be effective and are necessary to improve public confidence in vaccination.

## Data availability statement

The original contributions presented in the study are included in the article/Supplementary material, further inquiries can be directed to the corresponding authors.

## Ethics statement

The studies involving human participants were reviewed and approved by the Institutional Review Board of Guangzhou Medical University - approval: GZMU2020-07-0676. Written informed consent to participate in this study was provided by the participants' legal guardian/next of kin.

## Author contributions

LC analyzed and interpreted the data and completed the writing. ZL, XL, ZW, and LC took part in the investigation. JL, YD, and ZW made contributions to conceptualization, participant recruitment, and project management. JL, XL, TL, and LL guided the methodology and revised the manuscript. All authors read and approved the final manuscript.
