# Supplementary material for: Changes in COVID-19 vaccine hesitancy at different times among residents in Guangzhou, China
Source: Front Public Health. 2023 Jun 13;11:1164475. doi: 10.3389/fpubh.2023.1164475 (PMC10294687; doi:10.3389/fpubh.2023.1164475)
Supplement: Supplementary file 1 [file Data_Sheet_1.docx]

COVID-19 Vaccine Hesitation Behavior Questionnaire

**Guidance:** Dear friends, hello! This is the hotline of the Guangzhou Center for Disease Prevention 12320. To better carry out the work of COVID-19 vaccination and understand the public's attitude towards COVID-19 vaccination, we have prepared several questions. Please spare five minutes to help answer the following questions. Thank you!

First: basic information

1. Your age is: **________**
2. Your gender is:

1. Male 2. Female

1. Your mobile phone number is: **________**
2. Your current residence is:

1. Rural area 2. Urban area

1. Your education is:

1. ≤Primary school 2. Junior high school

3. high school 4. ≥Undergraduate

1. Your Monthly Income is:

1. <5,000 2. 5,000~10,000 3. >10,000

7. Your profession is:

1. Students 2. Teachers 3. Nannies 4. Waiters

5. Food and beverage industry 6. Medical personnel 7. Workers

8. Farmers 9. Staff 10. Civil servants or employees in public institutions

11. Unemployed 12. Other (Please specify) **________**

second: Do you have any vaccination-hesitating thoughts or behaviors? Which of the following fits your status:

Vaccine hesitancy refers to the mental state of refusing or resisting vaccination even when vaccination is available. It can also include behavioral vaccination but still having doubts or doubts.

What is your attitude towards COVID-19 vaccination?

1. fully accepted 2. accepted but unsure 3. partially accepted 4. delayed vaccination 5. partially rejected 6. rejected but unsure 7. completely rejected

Third: Have you been vaccinated against COVID-19?

1. Yes 2. No

How many doses of the COVID-19 vaccine have you received so far? ________

Fourth: Personal status

1. Do you have any underlying diseases (diabetes, hypertension, and other chronic diseases)?

1. Yes 2. No

1. How do you measure your health? (On a scale of 1 to 10, with "1" being unhealthy and "10" is very healthy) Score:
2. Have you had an unpleasant experience during the vaccination process?

1. Yes 2. No

1. Have you consulted a professional for the COVID-19 vaccine?

1. Yes 2. No

1. Do you think the emergence of mutant strains has an impact on vaccines?

1. Yes 2. No

1. Do you think getting the COVID-19 vaccine can reduce the symptoms of [COVID-19](javascript:;) in the future?

1. Yes 2. No

Vaccine Hesitation Influencing Factors Scale

| Item | strongly agree | agree | neutral | disagree | strongly disagree |
| --- | --- | --- | --- | --- | --- |
| Q1: Vaccination against Covid-19 is not effective in protecting me from Covid-19. |  |  |  |  |  |
| Q2: I'm healthy and don't need a Covid-19 vaccine. |  |  |  |  |  |
| Q3: I think it is better to prevent the Covid-19 infection by doing good personal protection. |  |  |  |  |  |
| Q4: The risk in our country is not high, and there is no need to be vaccinated against the Covid-19. |  |  |  |  |  |
| Q5: At present, the treatment methods are perfect, because of the Covid-19 vaccine is not so necessary. |  |  |  |  |  |
| Q6: Covid-19 vaccine remains risky as a new vaccine. |  |  |  |  |  |
| Q7: I have underlying diseases, so I don't want to get vaccinated against the Covid-19. |  |  |  |  |  |
| Q8: I am worried about the side effects of the Covid-19 vaccine. |  |  |  |  |  |
| Q9: I am concerned that the vaccine is a counterfeit or expired product. |  |  |  |  |  |
| Q10: I am concerned that the procedures of the vaccinators are not standardized. |  |  |  |  |  |
| Q11: I think vaccines are safe, but not very effective. |  |  |  |  |  |
| Q12: I don't know where to get the Covid-19 vaccine. |  |  |  |  |  |
| Q13: I don't have time to get the Covid-19 vaccine. |  |  |  |  |  |
| Q14: Vaccines are difficult to book. |  |  |  |  |  |
| Q15: The hesitant attitude of the medical staff about vaccines has had a great influence on me. |  |  |  |  |  |
| Q16: The hesitant attitude of colleagues or classmates about vaccines has had a great influence on me. |  |  |  |  |  |
| Q17: My family's hesitant attitude towards vaccines had a big impact on me. |  |  |  |  |  |
| Q18: Reports of vaccine hesitancy on social media have had a big impact on me. |  |  |  |  |  |

**Thank you for your participation. All investigations will be kept confidential.**
